# Adenitis, Acute and Chronic

**Published:** 1878-01

**Authors:** J. R. Larzelere


ART. II.—*Adenitis, Acute and Chronic.* By J. R. LARZELERE, M. D.

[Read at the October session, 1877, of the Muskingum County Medical Society.]

That we may have a more correct understanding of the subject, it seems necessary that your attention should be briefly called to the anatomical arrangement and physiology of the lymphatic system. The lymphatic apparatus is composed of a system of vessels, which if formed in a diagram similar to that of the blood vessels, presents the appearance of a cone, the summit of which joins the venous system in the subclavians, while the base or capillaries are in contact with the epithelium. The origin of the lymphatic system may be considered as closed by the epithelial membranes, save in the small intestines where the lymphatic net-work is separated from the epithelium by a blood net-work. Lymphatic spaces have been discovered situated around the smaller capillaries which have been named peri-vascular spaces. These spaces primitively have no limiting membrane, but gradually coalesce and form a small lymphatic vessel having a true limiting membrane. The plentiful supply of valves in this system of vessels makes it next to impossible for the contents to flow backward. A lymphatic ganglion is simply a plexus of lymphatic capillaries, anastomosed, and rolled up into a ball and held together by connective tissue. The physiology of the lymphatic system in brief, is that of carrying lymph and chyle, and in some inexplicable way the ganglion aid in the onward incubation of white corpuscles, all of which are emptied into the blood stream. Lymph in a broad sense is blood minus its red corpuscles; and chyle is lymph, plus a very large quantity of minutely divided neutral fat, and contains about eight or nine per cent of solid matter; say equal parts of fat and albumen and some fibrin, etc. The white corpuscles contain the aforesaid elements, which are known as the complex nitroginous fats.

Having thus briefly epitomised the anatomy and physiology of the lymphatic system, we are better able to perceive the effects of modification of structures which result from extrinsic or intrinsic causes. Hence, when a patient afflicted with either acute or chronic adenitis, is presented for professional opinion, we must not forget, that not the glands alone are affected, but, that the lymphatic capillaries and vessels are also implicated in the pathology. The acute form of adenitis is seldom, or ever idiopathic, but is almost invariably the result of angeioleucitis, accompanied with pain, swelling tenderness etc., followed by all the symptons of acute abscess. A bubo may be cited as a marked form of this affection; also, any ganglia of the economy which terminates in resolution or suppuration, consequent upon acute conditions.

The chronic or indiopathic form, whatever may be the cause, is always attended by a stasis of lymph, or coagulation of it, which so readily induces enlargement of the gland or glands, by obstructing the lymph channels, which so largely compose the ganglia. The normal course of the white corpuscles are arrested, as are the floating debris in bodies of water. When a large number of ganglia of a limb are thus affected, the course of the lymph through the vessels and glands is seriously interfered with, which soon results in œdema of the member affected. Hence, the exosmosis of the fluid elements of the lymph vessels into the areola tissue. In the diseased state known as Leukæmia, we have a manifest instance of the accumulation of white corpuscles, in some instances numbering as much as one to ten red corpuscles. With this physiological modification there can not be other than lymphatic ganglia and other forms of adenoid tissue enlargment.

The so called strumous diathesis of some individuals simply means favorable surroundings of the lymphatic capillaries, vessels and ganglia, which from trivial causes readily take on pathological states. Whatsoever may chance to retard the digestive function, is, to some extent, reflective upon the already de-depraved physiology of the absorbent system. Hence the primitive elements within the capillaries and vessels are abnormal, and in their onward course to the blood stream, cannot, by any probable prospective mutations, incubate into normal tissue. Therefore, the aggregation of the corpuscular elements in lymph vessels, and especially those entering into the formation of the ganglia. The coiled vessels of the glands are embolised with solids, while the non-solids of the lymph are osmosed into the surrounding cellular tissue which eventuates in extra vascular induration.

In further illustration of the subject, permit me to detail a short clinical report of a Mr. T———, aged about 30 years, who sought my counsel some seven years since. His state of health up to a period of two years preceding my first interview, he, himself considered good, however, I should judge he was one of those subjects which the books denominate strumous.

An examination revealed a large swelling, composed of an aggregation of enlarged cervical glands, on the left side of the neck, which well-nigh filled up the anterior and posterior triangles, especially the latter. The enlargement was so great, that he was unable to turn his head with any degree of comfort toward the left side. Isolated enlarged glands were also found imbedded in the opposite side. In common with most members of the profession, I considered myself equal to the emergency, and encouraged hopefulness in my ability to direct the therapeutics. And just here, I was open to the reflex comfort of the trite adage, "If ignorance is bliss, it is folly to be wise." I did not know much of the physiology at that time, this branch of science was just beginning to emerge from the labyrinths of the past ages of darkness. Hence, my inability to give the patient such counsel as best suited his condition.

Having faith, I directed such therapeutics, as recommended in the books, viz.: alterative tonics, and such other agents, as were supposed to act benignly on a pathological lympathic system. This course was persevered in for a reasonable time without any apparent good results. Feeling my faith in this method of therapeutics on the wane, I advised the use of the knife,—extirpation of the unsightly objects; which was confirmed by a member of the profession in the city, who was late from the gory fields of war. The patient was anesthetised with chloroform, and an incision commencing nearly on a level with the angle of the inferior maxillary, and extending almost to the clavicle; latteral flaps were formed by superficial dissection, and a score or more of enlarged glands removed, ranging in size, from that of a filbert, to a small walnut; still leaving the bottom of the wound, and adjacent parts, paved with enlarged ganglia. As these phenomena presented, it was thought the better part of valor to desist from any further removals. The patient regained consciousness in a reasonable time, and for a subsequent brief period, improved in his general health, and the reparative processes of the wound were benign, all of which infused hope in your reporter, that his patient might obtain much benefit from the treatment. But alas! for the lack of knowledge of his medical attendant and counselor in behalf of the physiology and pathology of the lymphatic system. For soon, phenomena were developed which made it apparent, that this system of vessels and glands could not even perform a shadow of function in the interest of health. Ædema of the lower extremities, and its rapid march to the abdominal cavity and chest, speedily placed the patient in the struggles of death.

In conclusion, what is the lesson to be learned from the above case, which I confess is so imperfectly detailed, yet, sufficiently so, for all students of the present to grasp the pathology. This was a case of chronic adenitis, I now believe, the pathology was confined to no topography of the economy, but that the entire lymphatic system was diseased. And as first seen by myself was beyond the reach of any known treatment, both then and the present. In all such cases it is the duty of the medical counselor to inform his patient that his condition is beyond the successful direction of professional knowledge, save, that which may be communicated that, if he or she are wise, they will so direct their thoughts and actions, as will prove most likely to add some degree of happiness to their prospective hereafter.

